# Incidental Finding of Adult Intussusception Following Screening Colonoscopy: A Case Report

**DOI:** 10.7759/cureus.114357

**Published:** 2026-08-11

**Authors:** Ryan LaCombe, Marc O Duverseau

**Affiliations:** 1 Surgery, Western Atlantic University School of Medicine, Freeport, BHS; 2 Surgery, Newton Medical Center, Newton, USA; 3 General Surgery, Newton Medical Center, Newton, USA

**Keywords:** adult intussusception, bowel obstruction, colonoscopy, intestinal lipoma, pathological lead point

## Abstract

Intussusception is a condition in which a portion of the bowel telescopes into an adjacent portion of the bowel. The present case describes adult intussusception following a routine screening colonoscopy. The patient had experienced episodic right lower quadrant pain but never sought medical care. He was ultimately found to have a small bowel mass that served as the lead point of the intussusception. This case underscores the importance of identifying the source of abdominal pain before performing a screening colonoscopy, regardless of how benign the abdominal pain may appear clinically.

## Introduction

Intussusception is defined as the telescoping of a proximal portion of the gastrointestinal (GI) tract into the lumen of an adjacent section of the GI tract [1]. While most cases of intussusception occur in the pediatric population, they can also occur in adults. Adult intussusception is a rare condition, accounting for 5% of all intussusception cases, 1% of bowel obstructions, and 0.003-0.02% of hospital admissions [1,2]. Up to 92% of adult intussusception cases are due to a pathological lead point, which ultimately results in surgical resection [1-3]. Such pathological lead points may be benign or malignant [3]. The presentation of intussusception in adults differs slightly from that in pediatric patients, who classically present with crampy abdominal pain, bloody diarrhea, and a palpable mass. Adults tend to present with abdominal pain and symptoms of bowel obstruction, which occur in over 70% of cases [4-6]. The literature describing instances of intussusception following colonoscopy is sparse. Furthermore, colonoscopy-related intussusception is not a commonly tracked complication of colonoscopy [7-9]. This paper describes a case of adult intussusception following colonoscopy that ultimately required surgical intervention.

## Case presentation

**Figure 1:**
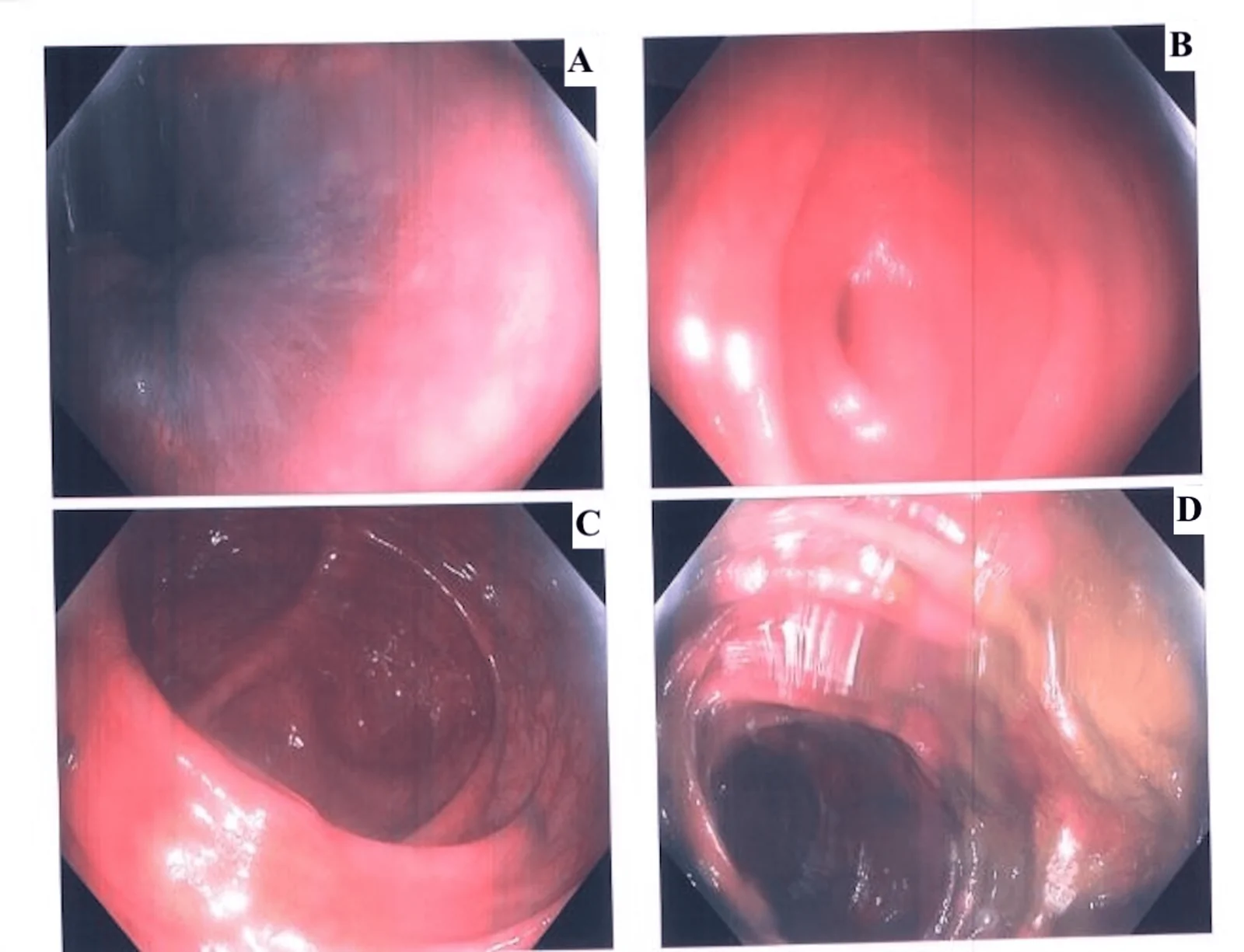
Colonoscopy images demonstrating the examined colonic mucosa (A) Internal hemorrhoids. (B) Healthy ascending colonic tissue. (C) Normal cecum. (D) Transverse colon with limited visualization.

A 65-year-old man with no significant past medical history presented to the hospital for a scheduled screening colonoscopy. He had a history of two adenomatous polyps at his most recent colonoscopy, for which he was recommended to undergo another screening colonoscopy three years later. He reported an infrequent history of mild right lower quadrant pain that always resolved spontaneously. The patient underwent colonoscopy, which was limited by poor visualization of the sigmoid, descending, and transverse colon; however, good visualization was achieved in the ascending colon and cecum. Internal and external hemorrhoids were noted, but no polyps were identified. Images from the colonoscopy are shown in Figure 1. Given the limited visualization and the patient’s history of adenomatous polyps, another colonoscopy was recommended in three years. The patient was discharged home without immediate complications.

**Figure 2:**
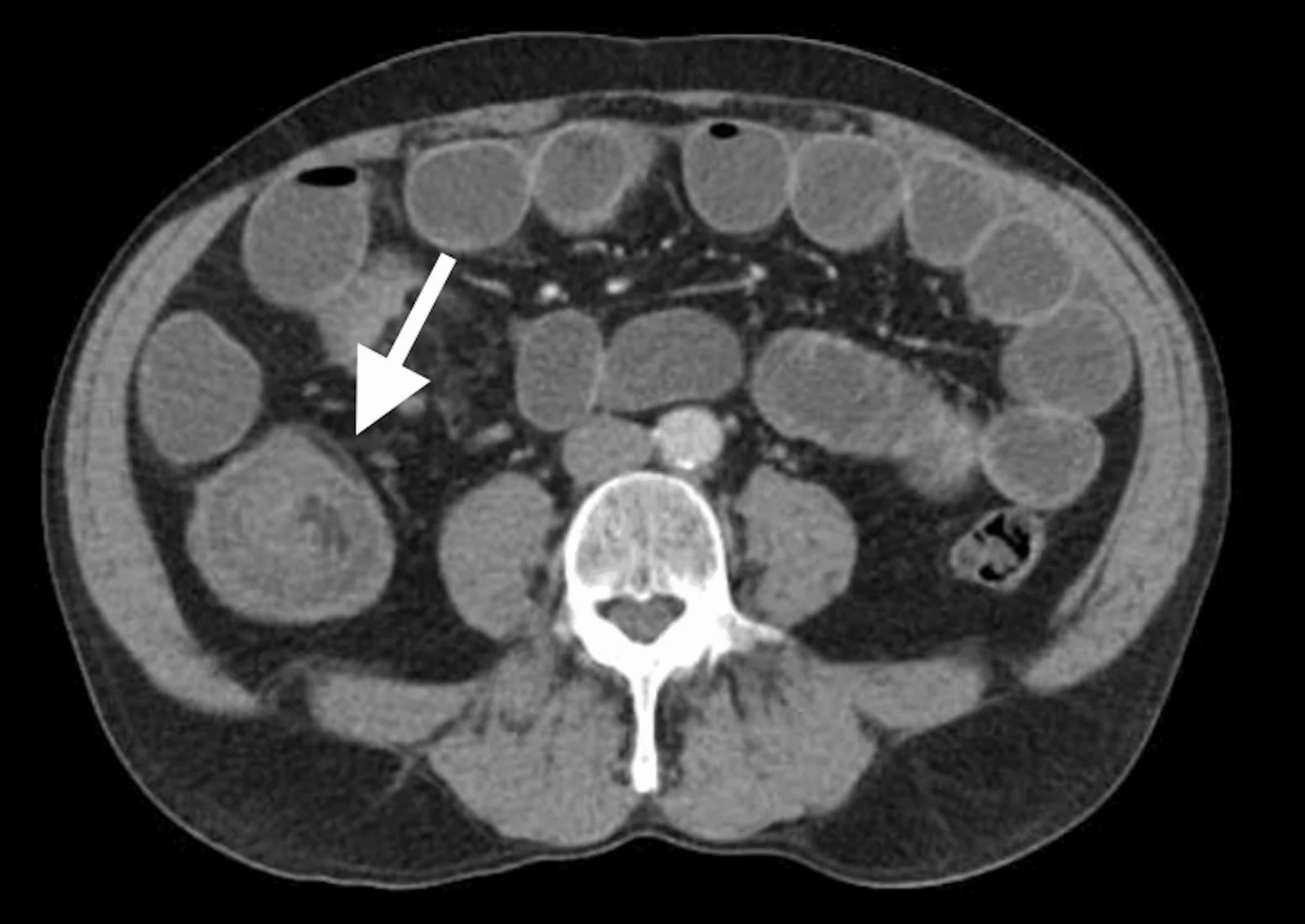
Axial view of the CT abdomen Pathognomonic telescoping ("target sign") is visualized on the left side of the image, as indicated by the arrow. CT, computed tomography

**Figure 3:**
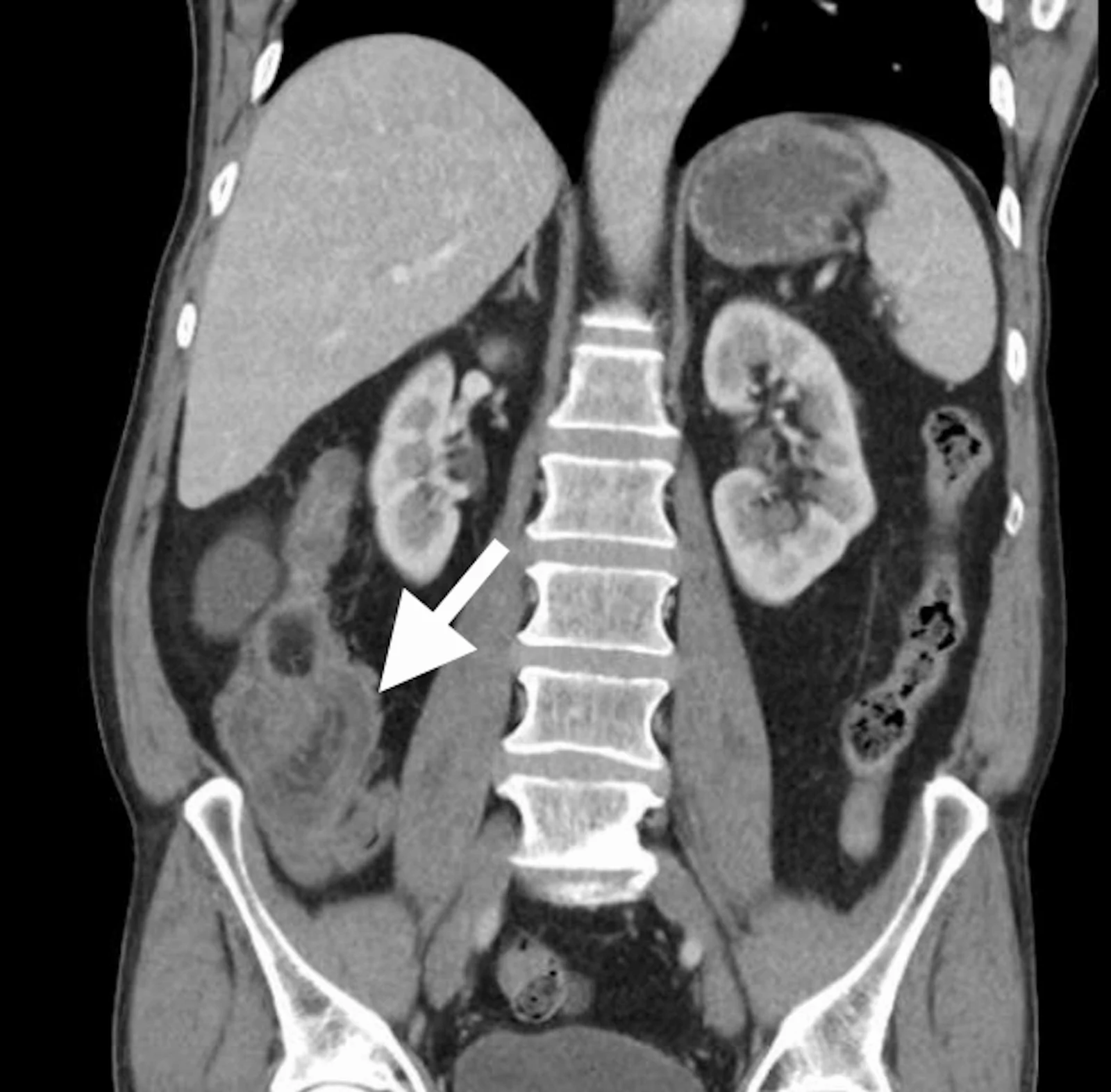
Coronal view of the CT abdomen Pathognomonic telescoping ("target sign") is visualized in the patient's right lower quadrant, as indicated by the arrow. CT, computed tomography

Twelve hours later, the patient presented to the emergency department with severe right lower quadrant pain. Initial laboratory workup showed a leukocyte count of 15,000; all other complete blood count (CBC) and complete metabolic panel (CMP) values were within normal limits. His CBC is shown in Table 1, and the CMP is shown in Table 2. Computed tomography (CT) of the abdomen showed an ileocolic intussusception with small bowel obstruction. These images are shown in Figure 2 and Figure 3.

**Table 1:** Complete blood count on admission WBC, white blood cell count; RBC, red blood cell count; Hgb, hemoglobin; Hct, hematocrit; MCV, mean corpuscular volume; MCH, mean corpuscular hemoglobin; MCHC, mean corpuscular hemoglobin concentration; RDW, red blood cell distribution width; Plt count, platelet count; MPV, mean platelet volume

Parameters	Patient value	Reference value
WBC	15	4.5-11.0 T/mm³
RBC	4.66	4.50-5.90 M/mm³
Hgb	14.5	13.5-17.5 g/dL
Hct	42.3	41-53%
MCV	90.8	80-100 µm³
MCH	31.1	26-34 µµg
MCHC	34.3	31-37 g/dL
RDW standard deviation	39	36.9-50.2 fL
Plt count	262	130-400 T/mm³
MPV	8.6	9.4-12.4 µm³

**Table 2:** Complete metabolic panel on admission BUN, blood urea nitrogen; GFR, glomerular filtration rate; AST, aspartate aminotransferase; ALT, alanine aminotransferase

Parameters	Patient value	Reference value
Sodium	140	136-146 mEq/L
Potassium	4.1	3.6-5 mEq/L
Chloride	104	98-107 mEq/L
Carbon dioxide	26	22-30 mEq/L
Anion gap	10	5-15 mEq/L
BUN	13	9-20 mg/dL
Creatinine	1	0.8-1.5 mg/dL
Estimated creat clear	83	>50 mL/min
GFR calculation	75	>60 mL/min
BUN/creatinine ratio	13	6-26 ratio
Glucose	106	75-110 mg/dL
Total bilirubin	0.5	0.20-1.30 mg/dL
Conjugated bilirubin	0	0.00-0.030 mg/dL
Unconjugated bilirubin	0.3	0.00-1.1 mg/dL
Incterus index	<2.0	0-7
AST	42	17-59 U/L
ALT	16	1-50 U/L
Alkaline phosphatase	107	38-126 U/L
Total protein	7.7	6.3-8.2 g/dL
Albumin	4.8	3.5-5.0 g/dL
Globulin	2.9	2.4-3.6 g/dL
Albumin/globulin ratio	1.7	1.1-2.2 ratio
Lipase	65	23-300 U/L
Calcium	10.2	8.4-10.2 mg/dL

The patient was subsequently taken to the operating room for a laparotomy. During evaluation of the bowel, the site of obstruction and intussusception was identified. Contrary to the imaging report, the defect was a distal ileoileal intussusception that had advanced through the ileocecal valve into the colon. The intussusception was manually reduced, and puckering of the distal ileum was noted. A hard, submucosal mass was identified in the distal ileum, serving as the lead point of the intussusception. An enlarged lymph node was also noted in the adjacent mesentery. Segmental small bowel resection with removal of the enlarged lymph node was performed. A side-to-side anastomosis completed the bowel intervention, and the remaining bowel showed no abnormalities following complete evaluation. The patient tolerated the procedure well and, following an uncomplicated hospital recovery, was discharged five days later.

The specimens were sent for pathological examination. Preliminary histologic examination suggested a submucosal and mural adipocytic lesion, which prompted the specimens to be sent to an outside laboratory for further testing beyond the capabilities of the local laboratory. Following examination, the lesion was determined to be a submucosal lipoma. Despite the relatively benign nature of this patient’s pathology, this case underscores the potential risks associated with seemingly simple screening procedures in the presence of unknown anatomic abnormalities.

Seemingly unrelated to the intussusception itself, the patient experienced abdominal pain four days after discharge. He experienced failure of the fascial sutures, requiring another operation. The bowel was inspected and showed three areas of induration and one area of serosal tearing, all of which were successfully imbricated. The patient tolerated the procedure well, recovered, and was discharged two days later. The patient attended his routine follow-up appointments and experienced no further complications.

## Discussion

The gold standard for diagnosing adult intussusception is contrast-enhanced CT of the abdomen [4]. Classic findings typically feature the diagnostic “target sign” or concentric rings of bowel and mesenteric fat [1,10]. Additional imaging modalities include ultrasound, which is technician dependent, and magnetic resonance imaging (MRI), which is typically used in combination with ultrasound [4]. Because adult intussusception is mostly caused by a pathological lead point that leads to bowel obstruction, surgical resection is the first-line treatment [2,4]. Depending on the malignancy status of the mass, the surgical approach is modified to address intussusception reduction and resection [1,3].

While the cause of this patient’s intussusception is unknown, one can reasonably postulate the combination of a small bowel mass (serving as lead point) and luminal pressure differences during colonoscopy created an optimal environment for this patient’s intussusception. This patient had noted episodes of right lower quadrant pain, which could have been due to mild, self-resolving cases of intussusception, or mild, self-resolving bowel obstruction due to the mass. Because the presence of a distal ileum mass alters the management of screening colonoscopy [11,12], one can assert further evaluation of right lower quadrant pain is warranted prior to colonoscopies. The main difference being attempted endoscopic biopsy of the lesion via terminal ileum intubation, if possible [13,14]. 

## Conclusions

The presence of right lower quadrant pain, however clinically benign, should prompt diagnostic imaging prior to performing a screening colonoscopy. Such imaging to identify a terminal ileal mass can help facilitate management and set expectations regarding post-colonoscopy recovery. Specifically, it may prompt the endoscopist to attempt terminal ileum intubation and biopsy of the lesion. Further, it would also be helpful to recognize that the risk of intussusception may be increased by the procedure.
